# Establishment of cross‐cradle hold technique combined with intensive breastfeeding counselling positively impacts the weight gain rate in early infancy

**DOI:** 10.1111/mcn.13529

**Published:** 2023-05-15

**Authors:** Rupal Dalal, Manish K. Fancy, Shalu Chaudhary, Marian Abraham, Sheila C. Vir, Sarthak Gaurav

**Affiliations:** ^1^ Centre for Technology Alternatives for Rural Areas Indian Institute of Technology‐Bombay & Shrimati Malati Dahanukar Trust Mumbai Maharashtra India; ^2^ Ministry of Health & Family Welfare Bhavnagar Gujarat India; ^3^ UNICEF & State Health System Resource Centre Gandhinagar Gujarat India; ^4^ Centre for Technology Alternatives for Rural Areas Indian Institute of Technology‐Bombay Mumbai India; ^5^ Public Health Nutrition and Development Centre New Delhi India; ^6^ Shailesh J. Mehta School of Management, Indian Institute of Technology‐Bombay Mumbai India

**Keywords:** 0–14 weeks of age, breastfeeding techniques, counselling, cross cradle hold, daily weight gain, underweight

## Abstract

A quasiexperimental field study was undertaken in 576 exclusively breastfed (EBF) infants from 0 to 14 weeks in Gujarat, India to assess the effect of the use of appropriate breastfeeding techniques on daily weight gain rate and on reducing the underweight rate in early infancy. The interventions were delivered through the existing health system and focused primarily on counselling pregnant women during antenatal and post‐natal sessions for ensuring ‘effective breastfeeding’ by adoption of the technique of ‘cross cradle hold’, appropriate breast attachment, emptying of one breast before shifting to the other along with conducting regular monitoring of infant's weight. The intervention care group (ICG) of 300 EBF infants were compared with 276 EBF infants in the control standard care group (SCG). The findings revealed that median weight gain per day between 0 and 14 weeks was significantly higher (*p* = 0.000) in ICG (32.7 g) as compared with SCG (28.05 g). The median weight‐for‐age *Z* at 14 weeks of age was also significantly higher in ICG compared with SCG (*p* = 0.000). Underweight prevalence was three times lower in ICG (5.3%) compared with SCG (16.7%) at 14 weeks of age. Infants in the ICG were noted to be 2.65‐fold more likely to achieve a weight gain of 30 g or more per day compared with infants in SCG. Nutrition interventions, therefore, must aim not only on mere promotion of EBF for up to 6 months but stress on ensuring EBF is ‘effective’ for optimum transfer of breastmilk through adoption of appropriate techniques, including cross‐cradle hold, by mothers.

## Key messages

- Breastfeeding, even if exclusive, can be suboptimal if the skills of mothers are not appropriate resulting in poor breastmilk transfer and poor intake of nutrients by a baby.
- Effective counselling on appropriate attachment to the breast, hold and positioning, is important to increase secretion of milk and optimise the transfer of breastmilk and thus improve infant nutritional status.
- This study shows that counselling mothers on cross cradle hold technique of breastfeeding during antenatal and post‐natal care periods combined with the frequent weighing of infants significantly escalates daily weight gain to over 30 g/day in 0–14 weeks of age.

## Graphical abstract

Intensive breastfeeding counselling with cross‐cradle hold starting from antenatal care with frequent weight monitoring visits of the baby reduces underweight prevalence at 3 months of age. Health Spoken Tutorials can be shown to healthcare workers as well as mothers to enhance their knowledge of breastfeeding skills.

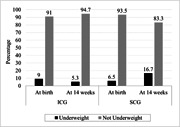

## 1 INTRODUCTION

Exclusive breastfeeding (EBF) provides all the required nutrients in the first 6 months of life and is one of the essential nutrition interventions recommended globally for addressing child undernutrition (Bhutta et al., 2013; World Health Organization [WHO], 2003). The rate of EBF of infants under 6 months of age in India is reported to be higher than the 47.4% observed in many lower‐middle‐income countries (Zong et al., 2021). Recent data reveals an increase in the EBF rate in India between 2015–2016 and 2019–2021 from 54.9% to 63.7% (International Institute for Population Sciences [IIPS] and Ministry of Health and Family Welfare [MoHFW], 2021a). Such an increase in EBF rates in India, however, does not correspond to a similar decrease in the undernutrition rates in the first 6 months of life—20.1% stunting and 26.7% underweight in 2015–2016 compared with 24.4% stunting and 28.5% underweight in 2019–2021 while the rate of wasting under 6 months infants declined from 31.9% in 2015–2016 to 27% in 2019–2021 (IIPS & MoHFW, 2017, 2022). Poor association of EBF with a decrease in early infancy undernutrition (height for age *Z*) is also evident from a cross‐sectional analysis of data from seven countries that revealed a significant negative association between EBF practice under 6 months infants and nutritional outcomes in two of the seven countries (Jones et al., 2014). On the other hand, a significant positive association has been reported between the total amount of breast milk consumed during EBF and nutritional status (weight‐for‐length and weight‐for‐age *z*‐scores) in exclusively breastfed infants aged 2–6 months (Urteaga et al., 2018). The importance of ensuring optimum milk transfer from mother to baby or ‘effective’ EBF appears critical in infants who are being exclusively breastfed in early infancy to positively impact on the total amount of breast milk consumed and nutritional status in the period 0–6 months. Poor breastfeeding practices during EBF have been attributed to the fact that only 7.5% of mothers are informed of the correct positioning and attachments (Parashar et al., 2015). Poor knowledge of appropriate techniques of breastfeeding, poor latching and poor suckling reduces breast milk output and lowers confidence of a mother that she is capable of producing adequate milk during EBF for her rapidly growing baby (Harsha & Kumar, 2017; Parashar et al., 2015; Santo et al., 2007). Lower output of breast milk due to such reasons strengthens the belief of a mother in breastmilk output being not adequate after a few weeks and results in a mother not adhering to the recommended EBF practice for the first 6 months of infancy (Ahluwalia et al., 2005). The need to counsel mothers to continue with EBF practice up to the first 6 months, therefore, needs to be combined with the provision of the required information and support to adopt appropriate breastfeeding techniques and ensure EBF is ‘effective’ with the optimum transfer of breastmilk (Mulder, 2006). Effective breastfeeding refers here to an interactive process between the mother and baby which results in transfer of breast milk from the mother's breast to the baby in a quantity and mode that is adequate to meet the needs of the infant. Baby's positioning, latching and suckling during EBF are therefore considered to be key characteristics of effective breastfeeding (Dalal et al., 2020; Kronborg & Vaeth, 2009; Mulder, 2006).

Empirical evidence on the effect of counselling that promotes adoption of effective breastfeeding practices on child nutritional status is limited. Interventions also do not focus adequately on breastfeeding techniques. Most breastfeeding studies have primarily concentrated on assessing the beneficial impact of breastfeeding counselling on outcomes that pertain to improving breastfeeding practices of mothers and not on the nutritional outcomes in infants (Adam et al., 2021; Shakya et al., 2017). Studies that have evaluated the impact of ‘effective’ breastfeeding with implications of the optimum transfer of breastmilk from mother to baby on the nutritional status in the first 6 months of life are limited to a study from Bangladesh and another one from Ethiopia. The Bangladesh study on low‐birth‐weight (LBW) infants focused on peer education and counselling on appropriate breastfeeding position and attachment and noted a decrease in underweight rates in LBW infants in the first 6 months (Haider & Saha, 2016). The peer‐support intervention in Ethiopia focused on appropriate breastfeeding positioning and latching and observed a significant impact of such an intervention on midupper arm circumference at 6 months but not on weight or length (Abdulahi et al., 2021). A study to understand the implications of the appropriate use of breastfeeding techniques by mothers who were exclusively breastfeeding their infants in India was considered essential to present evidence for investing in doable interventions for addressing the serious problem of a third of infants under 6 months being underweight and stunted in the country.

## 2 METHODS

### 2.1 Study regions settings

A community‐based field study, with a quasiexperimental design, was conducted between August 2020 and September 2021 in a preponderantly rural district of Banaskantha District of Gujarat in West India. In Gujarat state, 31.8% infants under 6 months are reported to be underweight, 32.3% wasted and 26.8% stunted in 2019–2021 (IIPS & MoHFW, 2021b). Selection of talukas or subdistrict administrative units (SDAU) of district Banaskantha, was done in consultation with the District Health Officer. Three SDAUs (Vadgam, Palanpur and Amirgadh) that were situated near the District Head Quarter (DHQ) were selected for the intervention care group (ICG) and four SDAUs (Tharad, Vav, Suigam and Bhabhar talukas) situated at a distance of approximately 100 km from the DHQ were selected for the standard care group (SCG). Selection of SDAUs for ICG and SCG took into consideration a safe physical distance between the two groups for preventing influence of the ICG interventions regarding promotion of the ‘cross cradle’ hold technique diffusing in the SCG region as well as ensured that the two selected study groups were comparable in terms of access to primary health care services including antenatal care (ANC), post‐natal care (PNC) services and presence of frontline workers of health (Accredited Social Health Activists [ASHAs] and Auxiliary Nurse Midwife [ANMs]).

### 2.2 Study design: Sample size and participants

For this quasiexperimental design study, the study subjects enroled were pregnant mothers in the third trimester of pregnancy, with the expected date of delivery between the period November 2020 and January 2021. The study period was up to 3 months postdelivery. The women who gave informed formal consent and provided assurance of not leaving their villages during the study period were enroled in the study. Institutional Ethics Approval and written informed consent were obtained from mothers before the enrolment in the study. The sample size calculation was based on power calculations using the available information on the proportion of exclusively breastfed children under 6 months of age in the study region. As per NFHS 4 data, 47.4% of children in Banaskantha district were reported to be exclusively breastfed while at state level (rural), 60.2% were exclusively breastfed (IIPS & MoHFW, 2016a, 2016b). Taking a proportion of 0.47 (P1) and 0.60 (P2) for breastfeeding for a power of study of 80%, and a level of significance of 5%, a sample size of 228 pregnant women per group was considered statistically appropriate. Due to the cultural practice of pregnant women visiting maternal homes for delivery, the rate of dropout during the study was estimated to be as high as 40% and the sample size was increased to 320 pregnant women for each of the two study groups, ICG and SCG.

Of the 57 Primary Health Centres (PHC) in the seven selected SDAUs, 40 PHCs were selected for the study. From each of the selected 40 PHCs, 16 women were selected and enroled for the study. Sixteen pregnant women per PHC is estimated to be on average about 4.9% of pregnant women that are registered at a PHC. Pregnant women from the two selected regions were randomly selected and enroled in ICG and SCG. The total sample size selected for the two groups in the study was 640 pregnant women. Of these, 576 mother–child pairs were enroled and followed up for the first 14 weeks study―300 mother–child pairs in ICG and 276 mother‐child pairs in SCG (Supporting Information: Appendix Figure 1). For babies' whose weight could not be taken for any reason, at 14th week, the weight at 15th or 16th or 13th week was considered.

**Figure 1:**
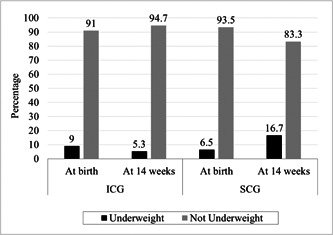
Underweight prevalence at birth and at 14 weeks. Underweight = weight for age *Z* < −2SD. ICG, intervention care group; SCG, standard care group.

### 2.3 Intervention design

The interventions were designed to be a part of the routine primary health care services for pregnant mothers under the National Health Mission (Ministry of Women & Child Development [MoWCD], 2010). For the ICG, counselling for effective EBF practice was undertaken during contact with mothers in the ANC and PNC periods. The medical officers (MOs) of PHCs, ANMs, and health supervisors were trained by master trainers at the district level and these conducted training of the ANMs and grassroots health workers referred to as ASHA at the PHC level. Counselling was facilitated by the use of specially developed four health‐spoken tutorials (HSTs). The focus of counselling was on adoption of ‘especially developed and tested Cross‐Cradle Hold’ technique for ensuring optimum transfer of breastmilk from mother to baby (Dalal & Agnihotri, 2021). The counselling sessions stressed (i) positioning (infant's stomach is close to the mother's stomach, head and body are in a straight line); (ii) latching (the mouth of the infant is wide open, the lower lip is curved outwards, the lower lip is placed on the border of the areola, asymmetrical latch, lips and chin are well embedded in the breast); (iii) contouring breast in order for deep attachment and (iv) physical methods to increase breastmilk supply and attention to emptying of one breast, objective examination and then only feeding by the second. Counselling sessions were undertaken by health grassroots workers/ASHAs during the routine ANC visits. In addition, the process of counselling included organising a screening of the HSTs and conducting demonstration sessions on cross‐cradle hold technique for breastfeeding with the use of models of a mother's breast made of a pliable material made by self‐group women and a newborn‐size plastic model. For such add‐on activities, a batch of three to four pregnant women in the last month of pregnancy was counselled by MOs and ANMs at the local PHCs. Mobility support for the PHC visit was facilitated by use of vehicles that are routinely provided by the government under ongoing schemes such as the Pradhan Mantri Matru Vandana Yojana and Janani Shishu Suraksha Karyakram (MoHFW, 2011; MoWCD, 2017). Additionally, following institutional delivery, mothers during PNCs were also counselled by ANMs and at times by MOs on a one‐to‐one basis on the techniques of effective EBF practices. Following discharge from the hospital, ANMs visited babies at the home level every week till a baby was 14 weeks. If for some reason, the baby was not visited at Week 14 then every attempt was made to visit the baby at 15th and 16th week or the weight at 13th week was considered. During these visits, the weight of infants was recorded using the government‐supplied weighing scales (Salter, Digital or Analog). During the follow‐up visits, information on breastfeeding assessment and anthropometrical measurements of the infant was also collected. The mothers were informed by ANMs of the progress in the weight gain of infants.

Pregnant women in the SCG or control group received routine counselling on promotion of EBF by ANMs and ASHAs as per the Home‐Based Care of New‐born (HBNC) guidelines of the Ministry of Health and Family Welfare, Government of India (MoHFW, 2014). As per HBNC guidelines, the community health workers (ASHAs) were trained and were expected to conduct a defined structured six home visits (on Days 3, 7, 14, 21, 28 and the 42nd day) in the first 6 weeks postdelivery. The tasks, as per guidelines, included counselling on maintaining the practice of EBF for a minimum period of 6 months, weighing of infants, counselling mothers newborn and post‐partum care, and clinical examination of mothers and newborns. The training package nor HBNC tasks made any reference to the cross‐cradle holding technique but emphasised appropriate latching to breast while breastfeeding. As per the HBNC policy, additional home visits were made by ASHAs to homes of only the high‐risk infants (i.e., preterm and low‐birthweight infants). However as per this study protocol, in addition to the HBNC recommended policy of weekly home visits, ASHAs were advised to continue with home visits beyond 6 weeks and up to the age of 14 weeks. During these add‐on visits, the tasks of health workers were limited to assessment of weight, informing mothers of the weight of infants as well as recording the practice of breastfeeding being followed by the mothers. However, during this period, unlike in the intervention group (ICG), no counselling on the significance or on the technique of breastfeeding was undertaken in the SCG. No special focus was given to counselling on cross cradle hold technique nor any special materials such as HSTs were provided to be used during the counselling sessions.

### 2.4 Data collection and analysis

The data for both ICG and SCG was collected using a pretested form in the web‐based application (Kobo). The baseline data for both ICG and SCG was collected by ANMs. The baseline data collection included basic information on mothers (education status, income of household, antenatal history, delivery and birth information). In addition, as described above, data on weekly weight records of infants and information on breastfeeding assessment and anthropometrical measurements of the infant was also maintained. Use of cross cradle technique by mothers was recorded for a full day and night and data were collected based on the mother's recall of the breastfeeding hold.

The data were primarily analysed to study the outcome of weight gain (g/day) between birth and 14 weeks. The secondary outcome focused on weight for age status at 14 weeks. The weight for age *Z* (WAZ) scores was generated using the software ENA for SMART 2020 which uses WHO, 2006 growth standards for calculating *z*‐scores (Emergency Nutrition Assessment, 2012). The statistical analyses were carried out using Stata (version 13.1). Intergroup medians were compared using nonparametric tests, Mann–Whitney *U* and Kruskal Wallis. Intergroup means were compared using the Student's **t**‐test. Proportions were compared using the *χ* ^2^ test. *p* Value < 0.05 was considered statistically significant.

## 3 RESULTS

As presented in Supporting Information: Appendix Figure 1, a total of 576 of the 640 randomized dyads participated in the study. A comparative analysis of the baseline findings available of 620 of the originally selected 640 dyads with 576 who finally participated in the study was assessed in terms of dyads assigned to each of the two groups and was noted to have no statistical difference. This analysis confirmed that the dropout subjects had no specific characteristics that would have influenced the study findings. The dropout of subjects seemed to be primarily due to the traditional practice of pregnant women shifting and staying in maternal homes for delivery and the early post‐natal phase.

In the study, data of a total of 576 infants from 0 to 14 weeks (ICG = 300 and SCG = 276) was analysed. The proportion of children who had a low birthweight, that is, weight below 2.5 kg was 13% in ICG and 8.3% in SCG (Supporting Information: Appendix Table 1). The two study groups had a similar proportion of children with a birthweight of 2.5 kg (12.7% ICG vs. 14.1% SCG), 2.6–2.9 kg (31.3% ICG vs. 34.1% SCG) and birthweight >2.9 kg (43% ICG vs. 43.5% SCG). There was no stillbirth or mortality reported in both groups. The availability and source of knowledge of EBF were significantly different for the ICG and SCG at the baseline (Supporting Information: Appendix Table 2). Only 75% of SCG mothers received information on breastfeeding techniques during the ANC period and among them, the primary source was family members (86.9%). SCG mothers had much lesser exposure to knowledge from sources such as healthcare workers. Only 13% of mothers in the SCG received information on breastfeeding techniques from healthcare workers. The rates of EBF at 14 weeks for both ICG (99.3%) and SCG (99.6%) were similar while the use of cross cradle hold for breastfeeding at the same age was significantly higher in the ICG as compared to SCG (ICG 97% vs. SCG 8.7%) (Supporting Information: Appendix Table 3).

**Table 1:** Comparison of weight gain (g/day) and improvement of WAZ between 0 and 14 weeks between intervention care group (ICG) and standard care group (SCG).

	ICG (*n* = 300)	SCG (*n* = 276)	*p* Value
Weight gain g/day between 0 and 14 weeks; Median (IQR)^a^	32.7 (28.9, 36.2)	28.05 (23.7, 33.3)	0.000
Weight gain g/day between 0 and 14 weeks; Mean (SD)^b^	32.14 (5.9)	28.15 (7.05)	0.000
Rates of weight gain g/day between 0 and 14 weeks; (%)^c^			
<30 g/day	32.70%	60.10%	0.000
≥30 g/day	67.30%	39.90%	
WAZ Gain between 0 and 14 weeks; Median (IQR)^a^	0.64 (0.01, 1.24)	0.15 (−0.6, 0.74)	0.000
WAZ gain between 0 and 14 weeks; Mean (SD)^b^	0.60 (0.94)	0.06 (1.03)	0.000
Underweight prevalence, that is, WAZ < −2SD at 14 weeks; (%)^c^			
Underweight	5.33%	16.67%	0.000
Not underweight	94.67%	83.33%	

Abbreviations: IQR, interquartile range; SD, standard deviation; WAZ, weight for Age *Z* score.

^a^ Mann–Whitney *U* test.

^b^ *t*‐test.

^c^ *χ* ^2^ test.

**Table 2:** Weight gain (g/day) between 0 and 14 weeks according to key characteristics in the intervention care group (ICG) and standard care group (SCG).

	ICG (*n* = 300)			SCG (*n* = 276)		
Key characteristics	Median (IQR)	Frequency	*p* Value	Median (IQR)	Frequency	*p* Value
*Mother's education* a						
Illiterate	33.4 (28.6, 36.7)	68	0.84	27 (23.2, 31.2)	96	0.12
Primary (1st–8th)	32 (28.9,36)	129		28.4 (24.3, 33.7)	150	
Secondary (9–10th)	33 (29, 35.8)	59		30 (24.5, 34.8)	24	
Higher Secondary and above	33.2 (30, 36)	44		32 (27.6, 35.7)	6	
*Gender* b						
Male	33.4 (29.7, 36.9)	166	0.000	30.3 (25.7, 35)	130	0.000
Female	31.4 (26.7, 35.1)	134		25.6 (21.6, 30.3)	146	
*Social category* a						
ST	33.7 (29.1,37)	65	0.4	23.6 (20, 25.7)	13	0.06
SC	32 (27.5, 36.6)	52		28.4 (23.5, 34.3)	64	
OBC	31.7 (28.5, 35.5)	156		28.3 (24.3, 32.5)	168	
General	33.5 (30.1, 36.1)	27		28.9 (25.5, 34.7)	31	
*Mother's religion* a						
Hindu	32.7 (29, 36.2)	262	0.46	27.8 (23.7, 33.3)	270	0.2
Others (Muslim, Sikh)	31.6 (27.8, 35.3)	38		30 (28.9, 34.5)	6	
*Birthweight* a						
<2.5 kg	32 (28.4, 36.1)	39	0.3	26.3 (23.5, 28.3)	23	0.13
2.5 kg	33.7 (29.7, 38.1)	38		29.1 (24.5, 32.7)	39	
2.6–2.9 kg	32.7 (29, 36.6)	94		28.2 (24.3, 33.7)	94	
>2.9 kg	32.1 (28.6, 35.8)	129		28.6 (23.4, 34.2)	120	
*Household size* a						
<4 members	31.5 (24, 34)	31	0.14	26.7 (24.5, 34)	37	0.35
4–6 members	32.7 (29.1, 36)	175		27.7 (23.7, 32.3)	156	
≥7 members	33.3 (29.1, 36.8)	93		29.8 (23.7, 34.5)	82	
*Monthly income* a						
<5000	31 (28.2, 35.3)	57	0.75	27.2 (23.7, 33.3)	93	0.67
5000–10,000	32.7 (29.4, 35.7)	138		28.6 (24.4, 33.4)	93	
>10,000–20,000	33.1 (27.5, 36.9)	68		24.9 (20.3, 35.4)	28	
>20,000–35,000	32.1 (25.5, 40)	15		28.6 (24.5, 34)	15	
>35,000	33.7 (31.1, 35.3)	11		28.8 (25.3, 32.4)	40	

Abbreviations: IQR, interquartile range; OBC, other backward class; SC, scheduled caste; ST, scheduled tribes.

^a^ Kruskal Wallis test.

^b^ Mann–Whitney *U* test.

**Table 3:** Factors associated with infant achieving at least 30 g/day weight gain between 0 and 14 weeks.

Variable	Crude OR (95% CI)	*p* Value	aOR (95% CI)	*p* Value
Study group				
SCG	1		1	
ICG	3.11 (2.2, 4.3)	0.000	2.65 (1.8, 3.8)	0.000
Gender of child				
Male	1		1	
Female	0.41 (0.29, 0.58)	0.000	0.40 (0.28, 0.58)	0.000
Birthweight				
≤2.5 kg	1		1	
>2.54 kg	1.17 (0.8, 1.7)	0.4	1.12 (0.74, 1.7)	0.57
Mother's education				
No education	1		1	
Primary	1.23 (0.83, 1.8)	0.28	1.17 (0.77, 1.77)	0.44
Secondary and above	2.59 (1.6, 4.1)	0.000	1.99 (1.18, 3.36)	0.009
Household size				
≤5 members	1		1	
>5 members	1.44 (1.03, 2.0)	0.03	1.44 (1.01, 2.06)	0.04
Monthly household income				
< Rs. 10,000	1		–	–
≥Rs. 10,000	1.41 (1.0, 1.9)	0.04		
First pregnancy status				
No	1		–	–
Yes	0.93 (0.64, 1.34)	0.7		

Abbreviations: aOR, adjusted odds ratio; ICG, intervention care group; OR, odds ratio; SCG, standard care group.

The median daily weight gain (g/day) between 0 and 14 weeks of age was assessed for both groups (Table 1). The weight gain g/day between 0 and 14th weeks of life (median [IQR]) was higher in the ICG (32.7 [28.9–36.2]) as compared with SCG (28.05 [23.7–33.3]). The difference between the two study groups, ICG and SCG, was significant (*p* = 0.000). The gain (median [IQR]) in WAZ at 14 weeks was higher in the ICG (0.64 [0.01–1.24]) as compared with SCG (0.15 [−0.6 to 0.74]). This difference was significant (*p* = 0.000). A weight gain of at least 30 g/day was achieved by 67.3% of children in the ICG against 39.9% in the SCG (*p* = 0.000). Underweight prevalence at 14 weeks was threefold higher in SCG compared with the ICG (16.7% vs. 5.3%) and this was statistically significant (*p* = 0.000) (Table 1, Figure 1). In the ICG, underweight prevalence decreased by 3.67% points between 0 and 14th week of life (decreased from 9.0% to 5.33%) whereas, in the SCG, the underweight prevalence increased by 10.15% points (increased from 6.52% to 16.67%) during the same period.

**Figure 2:**
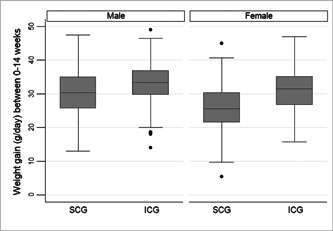
Median weight gain (g/day) between 0 and 14 weeks for intervention care group (ICG) and standard care group (SCG) according to gender.

The median daily weight gain (g/day) between 0 and 14 weeks was also analysed with reference to selected characteristics (Table 2). There were no significant differences in weight gain observed in both the study groups with reference to key socioeconomic characteristics including mother's education and monthly income. However, in both ICG and SCG, the median (IQR) weight gain was significantly lower in girls compared with boys (*p* < 0.05) (Table 2, Figure 2). At 14 weeks, the gain in weight observed against the birthweight records maintained at the time of institutional delivery was not significant for both ICG and SCG. In the case of ICG, the median (IQR) weight gain (g/day) from 0 to 14 weeks for LBW infants (32 g [28.4, 36.1]) was similar to children born with weight greater than 2.9 kg (32.1 g [28.6, 35.8]).

Table 3 presents findings from crude and multivariate logistic regression analysis where the dependent variable was whether infants achieved at least a daily weight gain (g/day) of 30 g/day between 0 and 14 weeks. In the adjusted multivariate model, infants in the ICG were 2.65 times more likely to achieve at least 30 g/day weight gain from 0 to 14 weeks compared with SCG (OR = 2.65, CI [1.8, 3.8], *p* < 0.05). In the adjusted multivariable model, the most significant determinants for achieving at least 30 g/day weight gain across the two study groups were the infant's gender and household size. Having a secondary or higher level of education was associated with an increased chance of the infants achieving at least 30 g/day weight gain between 0 and 14 weeks (aOR = 1.99, CI [1.18, 3.36], *p* < 0.05) compared with mothers with no education.

## 4 DISCUSSION

The need for prenatal and early‐life interventions for promotion of appropriate infant feeding practices has been considered essential (Victora et al., 2010). The current study provides evidence that merely counselling on EBF without providing support to follow the practice of appropriate holding of infants using cross cradle technique by mothers does not result in optimum breastmilk secretion. For ‘effective’ breastfeeding by mothers, counselling by health workers should be frequent, accompanied by regular monitoring of weight gain and ensuring mothers adopt the cross‐cradle technique. Moreover, trained health workers in the existing public health system should be advised to counsel mothers during the antenatal periods besides only the post‐natal periods to make a significant difference in influencing mothers in adoption of appropriate breastfeeding techniques. For promotion of effective breastfeeding practices, frequent counselling should be supported by use of innovative audio‐visual pedagogic tools such as the HSTs during the antenatal and post‐natal services while use of printed counselling materials should be encouraged during frequent follow‐up home visits. These support tools are proven to be convincing and effective and concur with the earlier report on the potential and benefits of combining video‐based interventions with face‐to‐face counselling in convincing mothers of the adoption of appropriate behavioural practices of breastfeeding in resource‐poor settings (Adam et al., 2021). The current study also reveals the significance of regular infant weight monitoring in influencing mothers who can ‘see’ the progressive weight increase and gain confidence in the adoption and continuation of the cross‐cradle hold method as an effective breastfeeding technique.

This experimental study presents evidence that EBF practice when the mother uses appropriate breastfeeding skills results in the optimum transfer of milk from mother to infants with a significant increase in daily weight gain and in reducing the rate of underweight in the first 3 months of life, irrespective of whether the infants were low birthweight or not. The findings emphasize that it is imperative that we focus not only on promoting the practice of EBF but also ensure that mothers are also informed of appropriate feeding techniques comprising cross‐cradle hold, proper latching and suckling and emptying of one breast before shifting feeding from the other one. The use of the cross‐cradle hold technique has been recommended earlier for preterm babies for prevention of undernutrition at an early age of before 3 months (Meier et al., 2007). The rationale is that babies in such early infancy have poor neck control and cross‐cradle hold supports the neck of the premature baby very well. Additionally, with the cross‐cradle hold, the baby is held close to the mother and the baby's head is supported well by the hand opposite the breast used to feed the baby. This helps with the extension of the neck while bringing the baby to the breast. The extension of the neck makes it easier for the baby to swallow breast milk. Also, in this hold, U shape contouring of the areola is recommended so it becomes easier for the baby to get a bigger part of the lower areola in the mouth (Dalal et al., 2020).

In concurrence with WHO growth standards that prescribe a median weight gain of 34.1 g/day for boys and 28.6 g/day for girls between 0 and 13th week (i.e., 3rd month), the current study findings also noted a comparable median weight gain between 0 and 14 weeks for boys (33.4 g/day) and for girls (31.4 g/day). A higher median weight gain noted in boys as compared with girls in both the intervention and control groups is in agreement with such gender differences in weight gain reported by WHO (WHO, 2006). The present findings on daily weight gain have implications for national policy or programme guidelines in India since the recommended weight gain in the national programme context is only 500 g/month or 17 g/day (MoWCD, 2018; National Institute of Public Cooperation and Child Development [NIPCCD], 2006). The basis or rationale of this recommended weight gain is not reported and could be attributed to an average achieved in the absence of cross cradle hold technique when breastfeeding is exclusive but milk transfer may not have been optimum. A daily weight gain guideline of as low as 17 g/day implies that a baby weighing 3 kg at birth would fall below the WHO 3rd percentile within 3 months (WHO, 2006). This is of concern. As evident from the current findings, there is an urgent need to revisit the policy and consider revising the recommended daily weight gain during EBF of the first 3 months to at least 30 g/day.

The intervention care package used in this study highlights the need to use appropriate training tutorials and convincing demonstration techniques by health workers and lactation counsellors in public and private health facilities for promoting exclusive and ‘effective’ breastfeeding using proper holding techniques. The study findings emphasise the importance of counselling on breastfeeding with the right frequency with the right messages and at the right ANC and PNC contact times when mothers are receptive to adopting the recommended breastfeeding behaviour practices. The study highlights the significance of counselling on the importance of following the practice of early initiation of breastfeeding, and EBF coupled with support to establish the technique of cross cradle hold method. Such breastfeeding counselling sessions should be an integral part of the third trimester ANC session and also of the postdelivery sessions in institutional set‐up as well as in the home environment when a mother is receptive to information but is unfortunately exposed to conflicting instructions. The findings indicate the urgent need to revisit the existing government guidelines on EBF (MoHFW, 2014, 2016) for considering inclusion of tasks of weekly weighing of infants, informing mothers of weight gain and providing support for establishing cross‐cradle technique and ensuring the focus is not only on the practice of ‘exclusive’ breastfeeding but also on ‘effective’ breastfeeding In this context, appropriate training of health workers and equipping them with support audio‐visual materials on ‘cross‐cradle hold’ technique is essential. Weekly weighing of infants in early infancy is crucial for imparting confidence to mothers for ensuring breastfeeding is not only exclusive but also effective for addressing the grave problem of undernutrition in the first 6 months of life. Moreover, it also needs to be recognised that adequate secretion of milk will also contribute to convincing mothers and in addressing the common prevalent incorrect community perception of inadequate secretion and insufficiency of breastmilk that is reported to be a primary barrier to continuation of EBF after the age of 2–3 months (Alive & Thrive, 2018).

### 4.1 Limitations

No attempt was made in the study to match women of the ICG and SCG for a number of confounding factors such as parity, maternal age and literacy, social category, socioeconomic status of households, gender preference and birthweight. The intervention package was implemented within the existing district public health infrastructure but the regular PHC services were at times adversely affected due to the Covid pandemic and affected the study. The possibility of results being influenced by diffusion of intervention package elements into SCG due to transfer of trained healthcare workers (MO, ANMs) from ICG to SCG during the study period or the counselling intervention from ICG having spilt over to SCG due to extensive use of social media messaging by frontline healthcare workers cannot be ruled out. This is also reflected in the results with 8% of mothers in the nonintervention group reporting to be following cross cradle technique for breastfeeding.

## 5 CONCLUSION

The study presents evidence that frequent and timely counselling on the practice of EBF combined with support to mothers in adoption of the appropriate breastfeeding technique of ‘cross cradle hold’ and regular monitoring of weight gain of infants 0–3 months facilitates in ‘effective ‘breastfeeding’. ‘Cross cradle hold’ technique results in better attachment and ensures a comfortable position for both mother and baby with full control of the baby's head position resulting in facilitating deep lower areola latching with effective milk transfer. Moreover, such a technique results in easy swallowing by the baby, higher production of the mother's milk and a higher milk transfer. Such ‘effective’ EBF has a significant positive impact on optimising transfer of breast milk from mothers to their infants and in influencing daily weight gain of about 30 g/day in the first 3 months and reducing the risk of underweight in infants at 3 months of age.

## AUTHOR CONTRIBUTIONS

Rupal Dalal, Manish K. Fancy and Shalu Chaudhary designed the study. Manish K. Fancy and Rupal Dalal led the implementation of the study. Marian Abraham analyzed the data and prepared the first draft. Sheila C. Vir, Rupal Dalal, Shalu Chaudhary and Sarthak Gaurav contributed to the results interpretation and critical revision of the manuscript. All authors reviewed and approved the final version of the manuscript.

## CONFLICT OF INTEREST STATEMENT

The authors declare no conflict of interest.

## 6 Ethical statement

The Institutional Ethics Committee of the Indian Institute of Public Health, Gandhinagar approved the study (Reviewed on Feb 19, 2020; TRC‐IEC No: 24/2020‐21).

## Supporting information

Supporting information.

## Data Availability

The data that supports the study findings would only be available from the corresponding author upon reasonable request.
