# Supplementary material for: Establishment of cross‐cradle hold technique combined with intensive breastfeeding counselling positively impacts the weight gain rate in early infancy
Source: Matern Child Nutr. 2023 May 15;19(4):e13529. doi: 10.1111/mcn.13529 (PMC10483946; doi:10.1111/mcn.13529)
Supplement: Supplementary file 1 — Supporting information. [file MCN-19-e13529-s001.docx]

**APPENDICES**

**Appendix Figure 1. Intervention Care Group (ICG) and Standard Care Group (SCG): Study sample size**


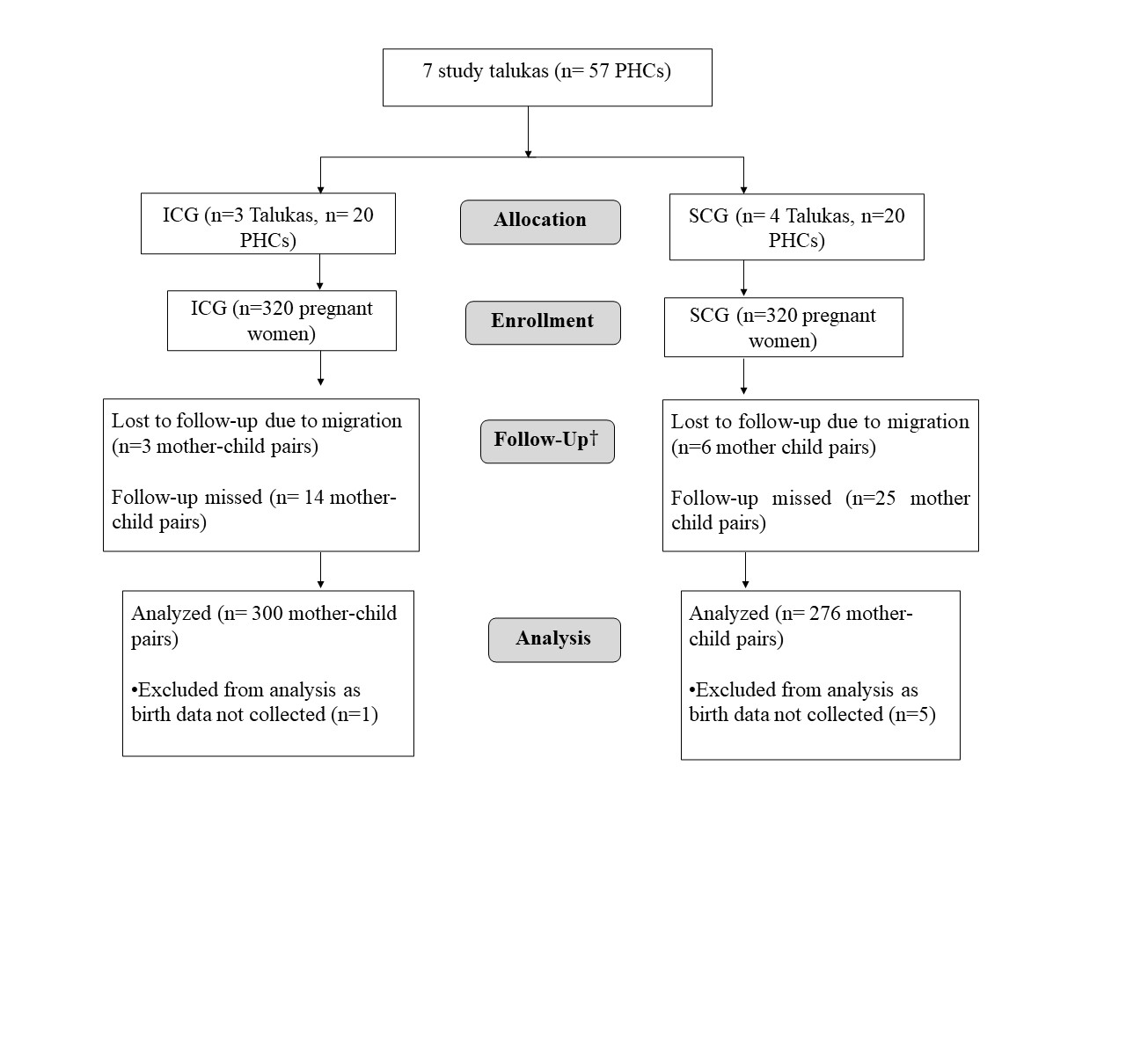


Abbreviations: PHC, Primary Health Centre

†Follow-up visit= visit during 14^th^ week however if 14^th^ week visit not done, visit in 15^th^ , 16^th^ or 13^th^ week was considered.

**Appendix Table 1. Baseline Characteristics of Intervention Care Group (ICG) and Standard Care Group (SCG)**

|  | **ICG (n=300)** | **SCG (n=276)** | ***P*-value** |
| --- | --- | --- | --- |
| Mother's education |  |  |  |
| Illiterate | 68 (22.6%) | 96 (34.8%) | 0.000 |
| Primary (1st-8th) | 129 (43%) | 150 (54.3%) |  |
| Secondary (9-10th) | 59 (19.6%) | 24 (8.7%) |  |
| Higher Secondary & above | 44 (14.6%) | 6 (2.2%) |  |
|  |  |  |  |
| Gender of Infant |  |  |  |
| Male | 166 (55.3%) | 130 (47.1%) | 0.048 |
| Female | 134 (44.7%) | 146 (52.9%) |  |
|  |  |  |  |
| Social Category |  |  |  |
| ST | 65 (21.7%) | 13 (4.7%) | 0.000 |
| SC | 52 (17.3%) | 64 (23.2%) |  |
| OBC | 156 (52%) | 168 (60.9%) |  |
| General | 27 (9%) | 31 (11.2%) |  |
|  |  |  |  |
| Mother's Religion |  |  |  |
| Hindu | 262 (87.3%) | 270 (97.8%) | 0.000 |
| Others (Muslim, Sikh) | 38 (12.7%) | 6 (2.2%) |  |
|  |  |  |  |
| Birth Weight |  |  |  |
| <2.5kg | 39 (13%) | 23 (8.3%) | 0.324 |
| 2.5kg | 38 (12.7%) | 39 (14.1%) |  |
| 2.6-2.9 kg | 94 (31.3%) | 94 (34.1%) |  |
| >2.9kg | 129 (43%) | 120 (43.5%) |  |
|  |  |  |  |
| Household size^†^ |  |  |  |
| <4 members | 31 (10.4%) | 37 (13.5%) | 0.519 |
| 4-6 members | 175 (58.5%) | 156 (56.7%) |  |
| >=7 members | 93 (31.1%) | 82 (29.8%) |  |
|  |  |  |  |
| Monthly Income^‡^ |  |  |  |
| <5000 | 57 (19.7%) | 93 (34.6%) | 0.000 |
| 5000-10000 | 138 (47.8%) | 93 (34.6%) |  |
| >10000-20000 | 68 (23.5%) | 28 (10.4%) |  |
| >20000-35000 | 15 (5.2%) | 15 (5.6%) |  |
| >35000 | 11 (3.8%) | 40 (14.9%) |  |

*Note*: Results based on Chi-Square Test. Data expressed in n (%).

Abbreviations: ST, Scheduled Tribes; SC, Scheduled Caste; OBC, Other Backward Class

^†^Household size sample size for SCG (n=275) and ICG (n=299).

^‡^Income sample size for SCG (n=269) and ICG (n=289).

**Appendix Table 2. Knowledge of breastfeeding techniques during ANC period for Intervention Care Group (ICG) and Standard Care Group (SCG)**

|  | **ICG (n=300)** | **SCG (n=276)** | ***P-*value** |
| --- | --- | --- | --- |
| Mother taught BF techniques during ANC |  |  |  |
| No | 0 (0%) | 69 (25%) | 0.000 |
| Yes | 300 (100%) | 207 (75%) |  |
|  |  |  |  |
| Source of mother's knowledge of BF techniques during ANC |  |  |  |
| Only family | 0 (0%) | 180 (86.9%) | 0.000 |
| Family & Healthcare workers | 1 (0.3%) | 2 (1%) |  |
| Only Healthcare workers | 299 (99.7%) | 25 (12.1%) |  |
|  |  |  |  |
| Other women in HH learnt techniques during PHC/Hospital visit |  |  |  |
| No | 18 (6%) | 239 (87.2%) | 0.000 |
| Yes | 282 (94%) | 35 (12.8%) |  |

*Note*: Results based on Chi-Square Test. Data expressed in n (%).

Abbreviations: ANC, antenatal care; BF, breastfeeding; HH, household; PHC, Primary Health Centre

**Appendix Table 3. Breastfeeding hold used at 14 weeks by Intervention Care Group (ICG) and Standard Care Group (SCG)**

|  | **ICG (n=300)** | **SCG (n=276)** | ***P*-value** |
| --- | --- | --- | --- |
| Breastfeeding hold during day |  |  |  |
| Only Cross Cradle | 272 (90.7%) | 19 (6.9%) | 0.000 |
| Cross cradle with other | 18 (6%) | 1 (0.4%) |  |
| Only Laidback | 0 (0%) | 1 (0.4%) |  |
| Only Side-line | 3 (1%) | 3 (1.1%) |  |
| Only Cradle | 5 (1.7%) | 246 (89.1%) |  |
| Only Football Hold | 0 (0%) | 1 (0.4%) |  |
| Two other holds | 2 (0.7%) | 5 (1.8%) |  |
|  |  |  |  |
| Breastfeeding hold at night |  |  |  |
| Only Cross Cradle | 136 (45.3%) | 13 (4.7%) | 0.000 |
| Cross cradle with other | 33 (11%) | 0 (0%) |  |
| Only Laidback | 7 (2.3%) | 5 (1.8%) |  |
| Only Side-line | 121 (40.3%) | 119 (43.1%) |  |
| Only Cradle | 1 (0.3%) | 120 (43.5%) |  |
| Two other holds | 2 (0.7%) | 19 (6.9%) |  |
|  |  |  |  |
| Breastfeeding during 24-hour period i.e both day & night |  |  |  |
| Only cross cradle both times | 132 (44%) | 9 (3.3%) | 0.000 |
| Cross Cradle with/without other hold at least once | 159 (53%) | 15 (5.4%) |  |
| One or more other holds both times | 9 (3%) | 252 (91.3%) |  |

*Note*: Results based on Chi-Square Test. Data expressed in n (%).
